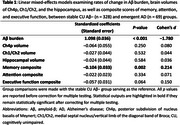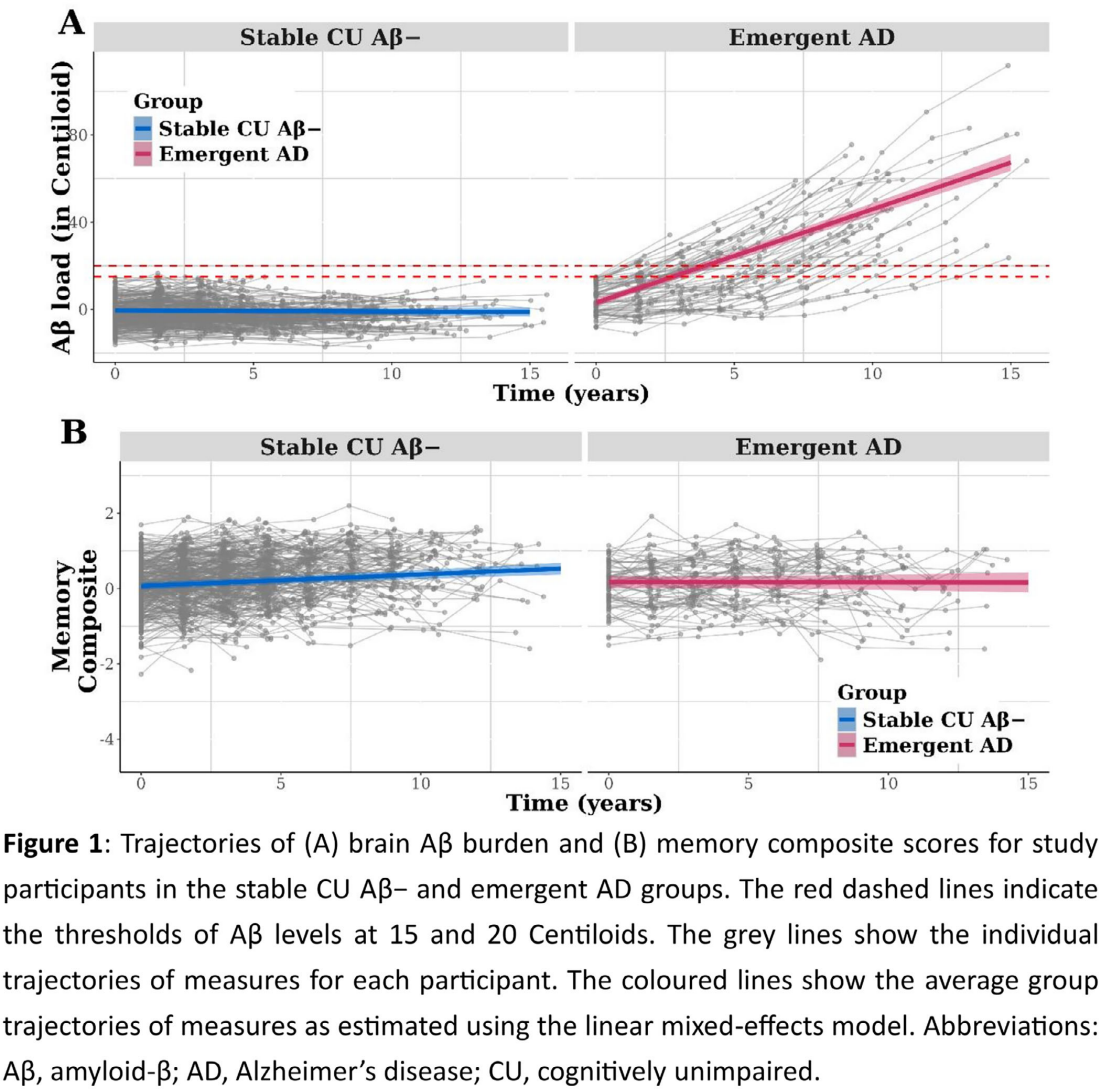# Correlations of amyloid accumulation, brain atrophy and cognitive decline in emergent Alzheimer's disease

**DOI:** 10.1002/alz70856_100789

**Published:** 2025-12-24

**Authors:** Ying Xia, Vincent Dore, Jurgen Fripp, Pierrick Bourgeat, Yen Lim, Simon M. Laws, Christopher J Fowler, Christopher C. Rowe, Colin L. Masters, Elizabeth J Coulson, Paul Maruff, for the AIBL study investigators

**Affiliations:** ^1^ CSIRO Health and Biosecurity, Australian E‐Health Research Centre, Brisbane, QLD, Australia; ^2^ School of Biomedical Sciences, The University of Queensland, Brisbane, QLD, Australia; ^3^ CSIRO Health and Biosecurity, Australian E‐Health Research Centre, Parkville, VIC, Australia; ^4^ Austin Health, Heidelberg, VIC, Australia; ^5^ Turner Institute for Brain and Mental Health, School of Psychological Sciences, Monash University, Melbourne, VIC, Australia; ^6^ Centre for Precision Health, Edith Cowan University, Joondalup, Western Australia, Australia; ^7^ Curtin Medical School, Curtin University, Bentley, Western Australia, Australia; ^8^ The Florey Institute of Neuroscience and Mental Health, The University of Melbourne, Parkville, Melbourne, VIC, Australia; ^9^ Austin Health, Melbourne, VIC, Australia; ^10^ Florey Institute of Neuroscience and Mental Health, University of Melbourne, Melbourne, VIC, Australia; ^11^ The Florey Institute of Neuroscience and Mental Health, The University of Melbourne, Parkville, VIC, Australia; ^12^ Queensland Brain Institute, The University of Queensland, Brisbane, QLD, Australia; ^13^ Cogstate Ltd., Melbourne, VIC, Australia

## Abstract

**Background:**

Amyloid‐β (Aβ) deposition and dysfunction of the cholinergic basal forebrain (BF) system are early pathological features in Alzheimer's disease (AD). Given the continuous nature of Aβ accumulation, the extent of the corresponding changes in brain structure and cognitive performance at the emergent stage of AD, characterized by subthreshold yet increasing Aβ burden, remains poorly understood. This study examined the relationships of Aβ accumulation, atrophy in the BF and hippocampus, and cognitive decline in initially Aβ− cognitively unimpaired (CU) older adults but later progressed to Aβ+, a transition defined as emergent AD.

**Method:**

CU individuals (*n* = 408, CDR=0, 71.6±5.3 years old, 58.6% female) from the Australian Imaging, Biomarkers and Lifestyle (AIBL) study who were initially Aβ− (< 15 Centiloids) and underwent repeated Aβ‐PET, MRI, and cognitive assessments every 18 months for up to 15 years were included. Linear mixed‐effects models were used to assess volumetric changes in BF subregions (Ch4p – posterior segment of the nucleus basalis of Meynert; Ch1/Ch2 ‐ medial septal nucleus and vertical limb of the diagonal band of Broca) and the hippocampus, as well as changes in domain‐specific cognitive composite scores, between groups stratified by their progression to Aβ+ (≥ 20 Centiloids).

**Result:**

Of the participants, 69 CU individuals progressed to Aβ+, indicating emergent AD, while 328 remained stable as CU Aβ−. As expected, the emergent AD group exhibited faster Aβ accumulation (Figure 1A). However, both groups showed no differences in rates of volume loss in the BF subregions and hippocampus (all *p* > 0.05; Table 1). Compared to the stable CU Aβ− group, the emergent AD group showed significantly greater declines in memory (*d* = 0.214, *p* = 0.002; Figure 1B), while no differences were observed in attention or executive function. Additionally, among stable CU Aβ− individuals, *APOE* ε4 carriers exhibited faster Aβ accumulation than non‐carriers (*d* = −0.188, *p* = 0.011), though no group differences were observed in brain volume or cognitive changes.

**Conclusion:**

During the emergent stage of AD, Aβ accumulation occurs without accompanying accelerated atrophy in the BF and hippocampus. However, the association between Aβ accumulation and episodic memory loss indicates that Aβ‐related neuronal dysfunction may begin prior to detectable volumetric changes.